# Exploring factors associated with a teacher’s self-efficacy in HIV-prevention education in Lusaka, Zambia

**DOI:** 10.1186/1475-9276-11-S1-A4

**Published:** 2012-01-23

**Authors:** Margaret Henning, Chunheui Chi

**Affiliations:** 1 Health Sciences, Keene State College, Keene, NH, 03431, USA; 2 International Health Program, Oregon State University, Corvallis, OR 97331, USA

## Background

Africa remains the epicenter of the global prevalence of Human Immunodeficiency Virus (HIV), which leads to Acquired Immunodeficiency Syndrome (AIDS) [1]. Evidence indicates school-based HIV-prevention programming is an effective step in protecting the general population from further HIV infection. Since HIV/AIDS is a major public health, social, economic and development challenge [2] the education sector is uniquely positioned within the community to support appropriate and sustainable change. The purpose of this study was to investigate the role of teachers as agents of change for HIV prevention, and to investigate key individual and social predictors of schoolteachers' self-efficacy as HIV-prevention educators in school settings in Lusaka, Zambia.

## Methods

Social Cognitive Theory guided this study, [3] which used an integrated qualitative and quantitative approach. An original cross-section of data was collected through self-reported survey interviews from schoolteachers in the Lusaka Province of Zambia. Schools in Lusaka Province were stratified according to type (private/church, community, and government) and were randomly selected in proportion to their number and type. Both quantitative and qualitative data was collected through survey and in-depth interviews with teachers from these schools, which provides triangulation of the work [4,5] and consideration for the theoretical underpinning.

## Results

In 2008, a sample of 720 teachers completed surveys within 123 sample schools that include all three types of schools, resulting a 91 percent response rate for teachers and 100 percent response rate for schools sampled (CI=95). Coefficients estimated from a linear regression model indicate that years of education, religion, adoption of HIV education, attitudes toward HIV, and gender norms were associated with a teacher’s self-efficacy toward HIV prevention in their respective school settings. Policy can be positioned to serve as a leverage point to address these factors and create sustainable change and support for teachers.

## Conclusion

Understanding teachers’ self-efficacy provides a holistic and integrated model to identify factors that can be addressed to promote successful school-based HIV/AIDS-risk-reduction programs and help in creating strategies to better facilitate teachers’ role as HIV educators within their cultural environment and classroom. There is a need for targeted capacity-building measures to focus on factors beyond a teacher’s control, and HIV/AIDS efforts across school types need to be mentored and supported continually. This work also suggests the need for further studies and insight into teachers’ beliefs about their skills as HIV-prevention educators while assessing the social environments that either enhance or hinder their work.
